# Jaccard/Tanimoto similarity test and estimation methods for biological presence-absence data

**DOI:** 10.1186/s12859-019-3118-5

**Published:** 2019-12-24

**Authors:** Neo Christopher Chung, BłaŻej Miasojedow, Michał Startek, Anna Gambin

**Affiliations:** 1 Institute of Informatics, Faculty of Mathematics, Informatics and Mechanics, University of Warsaw, Stefana Banacha 2, Warsaw, 02-097 Poland; 2 Institute of Mathematics, Polish Academy of Sciences, Jana i Jędrzeja Śniadeckich 8, Warsaw, 00-656 Poland

**Keywords:** Jaccard, Tanimoto, Binary similarity, Presence-absence, Co-occurrences, *P*-value, Primary 62F03; secondary 62-07

## Abstract

**Background:**

A survey of presences and absences of specific species across multiple biogeographic units (or bioregions) are used in a broad area of biological studies from ecology to microbiology. Using binary presence-absence data, we evaluate species co-occurrences that help elucidate relationships among organisms and environments. To summarize similarity between occurrences of species, we routinely use the Jaccard/Tanimoto coefficient, which is the ratio of their intersection to their union. It is natural, then, to identify statistically significant Jaccard/Tanimoto coefficients, which suggest non-random co-occurrences of species. However, statistical hypothesis testing using this similarity coefficient has been seldom used or studied.

**Results:**

We introduce a hypothesis test for similarity for biological presence-absence data, using the Jaccard/Tanimoto coefficient. Several key improvements are presented including unbiased estimation of expectation and centered Jaccard/Tanimoto coefficients, that account for occurrence probabilities. The exact and asymptotic solutions are derived. To overcome a computational burden due to high-dimensionality, we propose the bootstrap and measurement concentration algorithms to efficiently estimate statistical significance of binary similarity. Comprehensive simulation studies demonstrate that our proposed methods produce accurate *p*-values and false discovery rates. The proposed estimation methods are orders of magnitude faster than the exact solution, particularly with an increasing dimensionality. We showcase their applications in evaluating co-occurrences of bird species in 28 islands of Vanuatu and fish species in 3347 freshwater habitats in France. The proposed methods are implemented in an open source R package called jaccard (https://cran.r-project.org/package=jaccard).

**Conclusion:**

We introduce a suite of statistical methods for the Jaccard/Tanimoto similarity coefficient for binary data, that enable straightforward incorporation of probabilistic measures in analysis for species co-occurrences. Due to their generality, the proposed methods and implementations are applicable to a wide range of binary data arising from genomics, biochemistry, and other areas of science.

## Background

Analysis of species co-occurrences helps us understand ecological and biological relationships among species. Essentially, the presence (1) and absence (0) of species are surveyed in multiple biogeographic units (or bioregions) using fieldwork, imaging, sequencing, and other techniques. Then, the Jaccard/Tanimoto coefficient is one of the most fundamental and popular similarity measures to compare such biological presence-absence data. Given two presence-absence vectors ***y***_*i*_ and ***y***_*j*_ of length *m* that represent two different species, the Jaccard/Tanimoto similarity coefficient is the ratio of their intersection to their union, *T*(***y***_*i*_,***y***_*j*_)=***y***_*i*_∩***y***_*j*_/***y***_*i*_∪***y***_*j*_ [1, 2]. This quantification of overlaps allows us to quantify co-existence of species [3–6]. However, the Jaccard/Tanimoto coefficient lacks probabilistic interpretations or statistical error controls. Surprisingly, its statistical properties, hypothesis testing, and estimation methods for *p*-values have been inadequately studied. Here, we present a rigorous statistical test evaluating the similarity in presence-absence data, derive exact and asymptotic solutions, and introduce efficient estimation methods for significance of the Jaccard/Tanimoto similarity coefficient.

Generally, analysis of co-occurrences enables us to distinguish generalist species that survive in a broad range of environments from specialists that only thrive in a few localities [7, 8]. Alternatively, similarity between two localities – how two biogeographic units share an overlapping set of species – sheds light on the beta diversity that may arise from ecological processes over time [9–11]. There has been a long standing discussion on how to conduct association analysis for occurrences of species, including appropriate null models and evaluation techniques [12–17]. There are also specialized probabilistic approaches, including metrics related to the Jaccard/Tanimoto coefficient [18–21]. Yet, these studies rarely utilized statistical significance. Therefore, we investigated a hypothesis test using the Jaccard/Tanimoto coefficient that underlies or accompanies most of such association analyses.

The Jaccard/Tanimoto coefficient measuring similarity between two species has long been used to evaluate co-occurrences between species or between biogeographic units [3–5, 22–24]. Pioneering early works on probabilistic treatment of the Jaccard/Tanimoto coefficient assume that the probability of species occurrences is 0.5 [5, 22, 23]. These can be seen as special cases of our methods where both probabilities of ***y***_*i*_ and ***y***_*j*_ are set to 0.5. Recently, [24] and [25] proposed estimating *p*-values with combinatorics and hypergeometric distributions, respectively. We found that they may lead to inaccurate estimates. To provide a comprehensive statistical treatment, we have developed a suite of methods and estimation techniques for rigorously testing similarity between presence-absence data.

We derive a hypothesis test from the first principles using the Jaccard/Tanimoto coefficient. In the process, we propose an unbiased estimation of expectation and a centered Jaccard/Tanimoto coefficient that accounts for different probabilities of species occurrences. The negative and positive values of the centered Jaccard/Tanimoto coefficient naturally correspond to negative and positive association. We introduce an exact distribution of Jaccard/Tanimoto similarity coefficients under independence that is shown to provide accurate *p*-values. Because the exact solution for a large *m* is computationally expensive, we have developed two efficient and accurate estimation algorithms. We demonstrate their remarkable accuracy and computational efficiency in comprehensive simulation studies, where *p*-values and false discovery rates (FDRs) are evaluated. As applications, we evaluated co-occurrences of bird species from *m*=28 islands of Vanuatu and of fish species from *m*=3347 freshwater habitats in France.

All proposed methods are implemented in a statistical programming language R [26], available on the Comprehensive R Archive Network (https://cran.r-project.org/package=jaccard). We additionally provide an interactive web app (https://nnnn.shinyapps.io/jaccard). The implementations are efficient and general, such that the jaccard package can rigorously test similarity between binary data arising from genomics, biochemistry, and others.

## Methods

### Statistical model and test

Quantitative comparison of presence-absence data in ecology and biology plays a crucial role in evaluating species co-existences, biodiversities, and ecosystems. In particular, one may be interested in comparing how species are co-occurring in biogeographic units or how biogeographic units are occupied by certain species. Note that species are used generally to indicate groups of organisms under investigations, such as operational taxonomic units (OTUs); similarly, biogeographic units or bioregions could be distinct survey areas, islands, or habitats. We are interested in statistically testing similarity between a pair of presence-absence data.

**Fig. 1:**
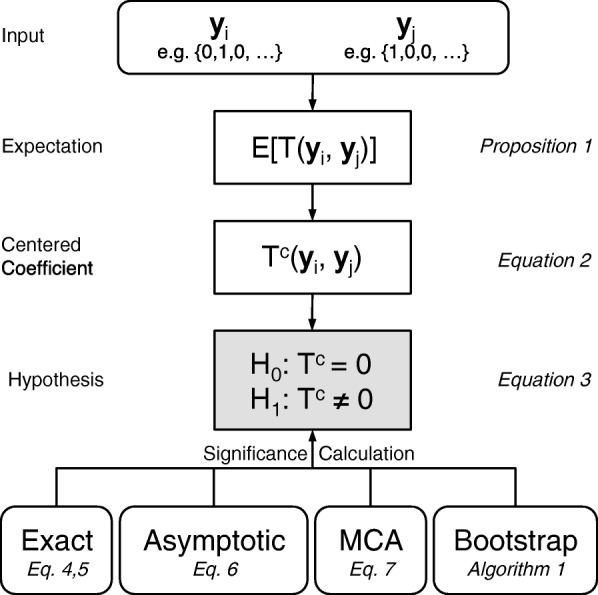
Flowchart of the proposed statistical methods and algorithms

Given two presence-absence vectors ***y***_*i*_ and ***y***_*j*_ of length *m*, we are interested in inferring whether they are significantly related. Consider presence (1) and absence (0) of two species are recorded at *m* biogeographic units. We measure their similarity by the ratio of their intersection to their union, *T*(***y***_*i*_,***y***_*j*_)=***y***_*i*_∩***y***_*j*_/***y***_*i*_∪***y***_*j*_. This is well known as the Jaccard/Tanimoto index or similarity coefficient [1, 2]. In order to utilize the Jaccard/Tanimoto similarity coefficient in a statistically rigorous manner, we propose a family of methods and algorithms (Fig. 1).

Under the null model of independence, ***y***_*i*_ and ***y***_*j*_ are assumed to be independent and identically distributed (i.i.d.). They are modeled by a Bernoulli distribution, with corresponding occurrence (i.e., success) probabilities *p*_*i*_ and *p*_*j*_∈[0,1]. Specifically, for *k*=1,…,*m*,***y***_*i,k*_∼_*i*.*i*.*d*._Bernoulli(*p*_*i*_) and ***y***_*j,k*_∼_*i*.*i*.*d*._Bernoulli(*p*_*j*_). Because this conventional definition is undefined if both binary vectors contain only zeros such that ***y***_*i*_∪***y***_*j*_=0, we refine the definition of Jaccard/Tanimoto coefficient

1 $$  T(\boldsymbol{y}_{i},\boldsymbol{y}_{j})= \left\{\begin{array}{ll} \frac{ \boldsymbol{y}_{i} \cap \boldsymbol{y}_{j} }{ \boldsymbol{y}_{i} \cup \boldsymbol{y}_{j}} & \quad\text{if } \boldsymbol{y}_{i} \cup \boldsymbol{y}_{j} \neq 0 \\ \frac{p_{i}p_{j}}{p_{i}+p_{j}-p_{i}p_{j}} & \quad\text{otherwise.} \end{array}\right.  $$

Following the definition of Jaccard/Tanimoto similarity coefficient in Eq. (1), we derive its expected value $\mathbb E[T(\boldsymbol {y}_{i},\boldsymbol {y}_{j})] =\frac {p_{i} p_{j}}{p_{i} + p_{j} - p_{i} p_{j}}$. Substantial deviation from the expected value signifies similarity. Note that the Jaccard/Tanimoto coefficient can also be defined in terms of a multinomial distribution with four categories and *m* trials (for example, representing *m* biogeographic units). Four categories arising from presence-absence data are *N*_1_=***y***_*i*_∩***y***_*j*_,*N*_2_=***y***_*i*_∩(1−***y***_*j*_),*N*_3_=(1−***y***_*i*_)∩***y***_*j*_ and *N*_4_=*m*−*N*_1_−*N*_2_−*N*_3_. From *p*_*i*_ and *p*_*j*_, probabilities of those four categories are *p*_*i*_*p*_*j*_,*p*_*i*_(1−*p*_*j*_),(1−*p*_*i*_)*p*_*j*_ and (1−*p*_*i*_)(1−*p*_*j*_), respectively. Putting them together, ***N***=(*N*_1_,*N*_2_,*N*_3_,*N*_4_) is distributed according to a multinomial distribution, Multi(*m,p*_*i*_*p*_*j*_,*p*_*i*_(1−*p*_*j*_),(1−*p*_*i*_)*p*_*j*_,(1−*p*_*i*_)(1−*p*_*j*_)).

#### **Proposition 1**

If ***y***_*i*_ and ***y***_*j*_ are independent, then

$$ \mathbb{E}(T(\boldsymbol{y}_{i},\boldsymbol{y}_{j})) =\frac{ p_{i} p_{j}}{p_{i} + p_{j} - p_{i} p_{j}}.   $$

#### **Proof 1**

First, we compute conditional expectation given *N*_1_+*N*_2_+*N*_3_. We observe that *N*_1_|*N*_1_+*N*_2_+*N*_3_ follows $\text {Bernoulli}(N_{1}+N_{2}+N_{3},\frac {p_{i}p_{j}}{p_{i}+p_{j}-p_{i}p_{j}})$. Hence, on set *N*_1_+*N*_2_+*N*_3_>0, we have

$${\begin{aligned} \mathbb{E}(T(\boldsymbol{y}_{i},\boldsymbol{y}_{j})|N_{1}+N_{2}+N_{3}) &= \mathbb{E}\left(\frac{N_{1}}{N_{1}+N_{2}+N_{3}}|N_{1}+N_{2}+N_{3}\right)\\ &= \frac{\mathbb{E}(N_{1}|N_{1}+N_{2}+N_{3})}{N_{1}+N_{2}+N_{3}}\\ &= \frac{\frac{p_{i}p_{j}}{p_{i}+p_{j}-p_{i}p_{j}}(N_{1}+N_{2}+N_{3})}{N_{1}+N_{2}+N_{3}}\\ &= \frac{p_{i}p_{j}}{p_{i}+p_{j}-p_{i}p_{j}}  \end{aligned}} $$

and on set *N*_1_+*N*_2_+*N*_3_=0, we have

$$ \mathbb{E}\left(T\left(\boldsymbol{y}_{i},\boldsymbol{y}_{j}\right)|N_{1}+N_{2}+N_{3}\right)=\frac{p_{i}p_{j}}{p_{i}+p_{j}-p_{i}p_{j}}   $$

Therefore,

$$\begin{array}{*{20}l}\mathbb{E}(T(\boldsymbol{y}_{i},\boldsymbol{y}_{j}))&=\mathbb{E}[\mathbb{E}(T(\boldsymbol{y}_{i},\boldsymbol{y}_{j})|N_{1}+N_{2}+N_{3})]\\ &= \frac{p_{i}p_{j}}{p_{i}+p_{j}-p_{i}p_{j}}\mathbb{P}(N_{1}+N_{2}+N_{3}=0) \\ & \hspace{2em} +\frac{p_{i}p_{j}}{p_{i}+p_{j}-p_{i}p_{j}}\mathbb{P}(N_{1}\,+\,N_{2}\,+\,N_{3}>0)\\&= \frac{p_{i}p_{j}}{p_{i}+p_{j}-p_{i}p_{j}}.  \end{array} $$

This allows us to define the centered Jaccard/Tanimoto coefficient as

2 $$  T^{c}(\boldsymbol{y}_{i},\boldsymbol{y}_{j}) = T(\boldsymbol{y}_{i},\boldsymbol{y}_{j}) - \mathbb E\left[T(\boldsymbol{y}_{i},\boldsymbol{y}_{j})\right]  $$

This accounts for expected values, naturally distinguishing negative and positive associations. Generally, we would like to measure the deviation of an observed coefficient from an expected value, instead of simply looking at a magnitude of an observed statistics. Furthermore, this centered coefficient may be scaled by variance in order to span a pre-defined range.

To evaluate whether ***y***_*i*_ and ***y***_*j*_ are independent, a following statistical hypothesis testing is performed:

3 $$\begin{array}{*{20}l}   H_{0} &: T^{c}\left(\boldsymbol{y}_{i},\boldsymbol{y}_{j}\right) = 0 \\ H_{1} &: T^{c}\left(\boldsymbol{y}_{i},\boldsymbol{y}_{j}\right) \neq 0.  \end{array} $$

The null hypothesis *H*_0_ is that the centered Jaccard/Tanimoto coefficient equals zero. Note that this is equivalent to that the conventional (uncentered) Jaccard/Tanimoto coefficient equals an expected value under independence. Therefore, although we propose and use the centered coefficient, this hypothesis testing is attributed to both uncentered and centered versions. Then, a *p*-value indicates a probability of observing a coefficient equal to or more extreme than an observed coefficient under the null hypothesis.

### Distribution of the jaccard/Tanimoto coefficient

To obtain its *p*-value, we derive the distribution of Jaccard/Tanimoto coefficient under the null hypothesis. In terms of ***N***=(*N*_1_,*N*_2_,*N*_3_,*N*_4_), the Jaccard/Tanimoto coefficient can be expressed as

$$ T(\boldsymbol{y}_{i},\boldsymbol{y}_{j})= \left\{\begin{aligned} \frac{N_{1}}{N_{1}+N_{2}+N_{3}} &\,\,\quad\text{if}\,\, N_{1}+N_{2}+N_{3}>0\\ \frac{p_{i}p_{j}}{p_{i}+p_{j}-p_{i}p_{j}}&\,\,\quad\text{otherwise.}  \end{aligned}\right.  $$

When *p*_*i*_ and *p*_*j*_ are known, the *p*-value is given by $\mathbb {P}(K_{T^{c}})$ where

4 $$ {\begin{aligned} K_{T^{c}}&=\left\{(N_{1},N_{2},N_{3},N_{4})\colon \left|\frac{N_{1}}{N_{1}+N_{2}+N_{3}} \right.\right.\\& \left. \left.- \mathbb E \left[T(y_{i},y_{j}){\vphantom{\frac{N_{1}}{N_{1}+N_{2}+N_{3}}}}\right]\right|\geq |T^{c}|\right\}\;. \end{aligned}}  $$

However, in practice, probabilities *p*_*i*_ and *p*_*j*_ are usually unknown. Therefore, we define the centered Jaccard/Tanimoto coefficient by $\hat T^{c} = T- \frac {\hat p_{i}\hat p_{j}}{\hat p_{i} +\hat p_{j}- \hat p_{i}\hat p_{j}}$, where $\hat p_{i} =\frac {\sum {\boldsymbol {y}_{i}}}{m}, \hat p_{j} =\frac {\sum {\boldsymbol {y}_{j}}}{m}$ are standard estimators of *p*_*i*_ and *p*_*j*_ respectively. Plug-in estimates of $\mathbb E[T(y_{i},y_{j})]$ into Eq. (4) will result in conservative behaviors, since we estimate the probabilities on the same sample that we want to perform the test. Then, the estimates of expectation are biased toward the observed value of Jaccard/Tanimoto coefficient. To overcome this bias, we estimate probabilities *p*_*i*_ and *p*_*j*_ for each configuration (*N*_1_,*N*_2_,*N*_3_,*N*_4_) separately.

So in this case, the critical region is defined as follows

5 $$ {\begin{aligned} K_{\hat T^{c}}=\left\{(N_{1},N_{2},N_{3},N_{4})\colon \left |\frac{N_{1}}{N_{1}+N_{2}+N_{3}} \right. \right. \\ \left. \left.-\frac{\tilde p_{i}\tilde p_{j}}{\tilde p_{i}+\tilde p_{j} -\tilde p_{i}\tilde p_{j}}\right|\geq |\hat T^{c}|\right\}\;, \end{aligned}}  $$

where $\tilde p_{i} = \frac {N_{1}+N_{2}}{m}$ and $\tilde p_{j} = \frac {N_{1}+N_{3}}{m}$.

Because the exact distribution is computationally expensive (see *Results* for comparison), we introduce an asymptotic approximation when *m*→*∞*. It may be useful when dealing with very large binary data, where computational power is a bottleneck. Denote by *q*_1_=*p*_*i*_*p*_*j*_ the probability that both ***y***_*i*_ and ***y***_*j*_ have ones, and by *q*_2_=*p*_*i*_+*p*_*j*_−2*p*_*i*_*p*_*j*_ the probability that only one of two vectors has one. Similarly, $\hat q_{1}$ and $\hat q_{2}$ are defined with the plug-in estimators. As *m*→*∞*, we can estimate the variance:

#### **Proposition 2**

If ***y***_*i*_ and ***y***_*j*_ are independent then

$$ \sqrt{m}T^{c}(\boldsymbol{y}_{i},\boldsymbol{y}_{j})\to\mathcal{N}(0,\sigma^{2})   $$

as *m*→*∞*, where

$$ \sigma^{2}=\frac{q_{1}q_{2}(1-q_{2})}{(q_{1}+q_{2})^{3}}.   $$

#### **Proof 2**

Theorem 14.6 of [27] states that

$$ \sqrt{m}\left((N_{1},N_{2}+N_{3})/m-(q_{1},q_{2})\right)\to\mathcal{N}(0,\Sigma)   $$

where

$$ \Sigma= \left[\begin{array}{cc} q_{1}(1-q_{1})& -q_{1}q_{2}\\ -q_{1}q_{2} & q_{2}(1-q_{2})  \end{array}\right].  $$

Then, we define function $g(x_{1},x_{2})=\frac {x_{1}}{x_{1}+x_{2}}$ and apply the delta method. So, we get

$$ \begin{aligned} &\sqrt{m}\left(T(\boldsymbol{y}_{i},\boldsymbol{y}_{j})-\frac{q_{1}}{q_{1}+q_{2}}\right)\\&\quad \to\mathcal{N}(0,\nabla g(q_{1},q_{2})\Sigma\nabla g(q_{1},q_{2})^{T}).  \end{aligned}  $$

The gradient of *g* is

$$ \nabla g(x_{1},x_{2})=\left[\frac{x_{2}}{(x_{1}+x_{2})^{2}},\frac{-x_{1}}{(x_{1}+x_{2})^{2}} \right].   $$

Finally, after simplification, we obtain

$$ \nabla g(q_{1},q_{2})\Sigma\nabla g(q_{1},q_{2})^{T}=\frac{q_{1}q_{2}(1-q_{2})}{(q_{1}+q_{2})^{3}}.   $$

In practice, probabilities *p*_*i*_ and *p*_*j*_ are unknown and need to be estimated. Recall that $\hat p_{i}=\frac {\# \{y_{ik}=1\}}{m}$ and $\hat p_{j}=\frac {\# \{y_{jk}=1\}}{m}$. We define $\hat q_{1}$ and $\hat q_{2}$ by replacing in definition of *q*_1_ and *q*_2_ true probabilities *p*_*i*_ and *p*_*j*_ by its estimators. So based on Proposition 2 we are able to approximate *p*-values as follow:

6 $$ 2\phi\left(\frac{\sqrt{m}}{\sigma}\left(T(\boldsymbol{y}_{i},\boldsymbol{y}_{j})-\frac{\hat q_{1}}{\hat q_{1}+\hat q_{2}}\right)\right)-1\;,  $$

where $\phi = \frac {1}{\sqrt {2\pi }} \int _{- \infty }^{x} e^{-x^{2}/2}dx$ is a standard Gaussian cumulative distribution function (CDF).

### Measure concentration algorithm

The distribution of the centered Jaccard/Tanimoto coefficient can be expressed in terms of the multinomial distribution. However, evaluating a significance test based on this representation requires exhaustive computations. It needs summation over all possible states of the multinomial distribution. For the centered Jaccard/Tanimoto coefficient between *y*_*i*_ and *y*_*j*_, we need to compute probability of event $ K_{\hat T^{c}}$ defined by Eq. (5).

This can be quickly and accurately estimated by the measure concentration algorithm (MCA) with a known error bound [28]. For every *ε*>0, we will construct *I*_*ε*_, a set of (*N*_1_,*N*_2_,*N*_3_,*N*_4_) with *N*_1_+*N*_2_+*N*_3_+*N*_4_=*m*, such that $\mathbb P (N_{1},N_{2},N_{3},N_{4})\in I_{\varepsilon } \geq 1-\varepsilon $. Given the set *I*_*ε*_, we have following bounds

$$ \begin{aligned} p^{L}_{\varepsilon}(\hat T^{c}) &= \mathbb P\left(K_{\hat T^{c}}\cap I_{\varepsilon}\right)\leq \mathbb P\left(K_{\hat T^{c}}\right)\\&\quad \leq \mathbb P\left(K_{\hat T^{c}}\cap I_{\varepsilon}\right)+\varepsilon= p^{U}_{\varepsilon}(\hat T^{c}).  \end{aligned}  $$

In addition, $p^{U}_{\varepsilon }(\hat T^{c})-p^{L}_{\varepsilon }(\hat T^{c})=\varepsilon $.

The idea behind the algorithm is that a multinomial distribution concentrates around its mode. Two possible states ***N***=(*N*_1_,*N*_2_,*N*_3_,*N*_4_) and $\boldsymbol N^{\prime }=(N_{1}^{\prime },N_{2}^{\prime },N_{3}^{\prime },N_{4}^{\prime })$ are neighbors, ***N***∼***N***^***′***^, if $\sum _{i=1}^{4}|N_{i}-N^{\prime }_{i}|=2$. This means that ***N***^′^ can be obtained from ***N*** by moving one element to a different class. We construct the set *I*_*ε*_ as follows.

At the onset, *I*_*ε*_ contains only the mode of multinomial distribution. We find the mode by a simple hill climbing algorithm, which starts with a state close to the mean of the multinomial distribution and follows the direction of increasing probability until the maximum is reached. Because of unimodality, it is indeed a global maximum. In the next steps, we add the neighbors of states which were previously visited. The procedure is repeated until the total probability of set *I*_*ε*_ reaches the desired value 1−*ε*. The details of the above method can be found in [28]. We construct the set *I*_*ε*_ and we estimate the *p*-value by

7 $$ {\begin{aligned} p^{L}(\hat T^{c}) = & \sum_{\boldsymbol N \in I_{\varepsilon}} \mathbf{1}\left(\left|\frac{N_{1}}{N_{1}+N_{2}+N_{3}} - \frac{\tilde p_{i}\tilde p_{j}}{\tilde p_{i} +\tilde p_{j}-\tilde p_{i}\tilde p_{j}}\right|\geq |\hat T^{c}|\right)\\ & \mathbb{P}(N_{1},N_{2},N_{3},N_{4}). \end{aligned}}  $$

### Bootstrap procedure

The bootstrap procedure has gained mainstream popularity for its wide applicability and statistical treatments [29]. Creating an empirical distribution of null statistics allows for a flexible and robust estimation of *p*-values and related statistics. We show how to use the resampling with replacement to obtain statistical significance of *T*^*c*^(***y***_*i*_,***y***_*j*_). Particularly, resampling with replacement ***y***_*i*_ and ***y***_*j*_, separately, breaks any potential dependency. This allows us to calculate an empirical distribution of Jaccard/Tanimoto coefficients under the null hypothesis:


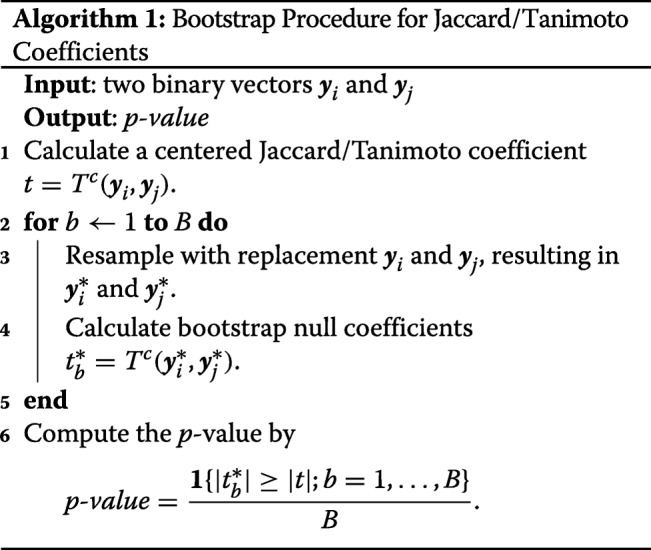


The expectation of Jaccard/Tanimoto coefficients is estimated directly from resampled vectors $\boldsymbol {y}^{*}_{i}$ and $\boldsymbol {y}^{*}_{j}$, that are effectively independent. Therefore, each iteration provides randomness, which helps avoid a bias related to using an estimated expectation based only on observation. Previously, there are early works in Monte Carlo procedures [14, 30] and published statistical tables for assessing randomness in species co-occurances [22, 23]. However, earlier works have assumed that a probability of occurrences is 0.5 regardless of species or biogeographic units. Permutation methods based on conventional uncentered coefficients are available in R packages, whose operating characteristics are not described in details [31, 32].

The resolution of the empirical null distribution depends on *B*, where the larger *B* will result in more precise estimation of *p*-values. Although the choice of *B* would likely be dictated by *n* and *m*, as well as available computational power, we recommend setting *B* to at least 5-10 times of *m*. In our simulation studies, the total bootstrap iterations is set to *B*=5×*m*, which are shown to be both accurate and fast. When comparing a very large set of species or OTUs, it may be helpful to pool null statistics to increase the *p*-value resolution and speed up the computation.

## Results and discussion

### Simulation studies

We have developed statistical methods and algorithms to obtain statistical significance of Jaccard/Tanimoto similarity coefficients for biological presence-absence data. Beyond deriving the exact solution, we introduce the measurement concentration algorithm (MCA) and bootstrap method. We characterize their operating characteristics by comprehensive simulation studies where a wide range of parameters for presence-absence datasets are considered. Our goal is to maintain theoretically correct behaviors of *p*-values. Null *p*-values corresponding to *H*_0_ are evaluated against a Uniform(0,1) distribution. False discovery rates (FDRs) are directly estimated from *p*-values produced by our methods to demonstrate an overall error control.

**Fig. 2:**
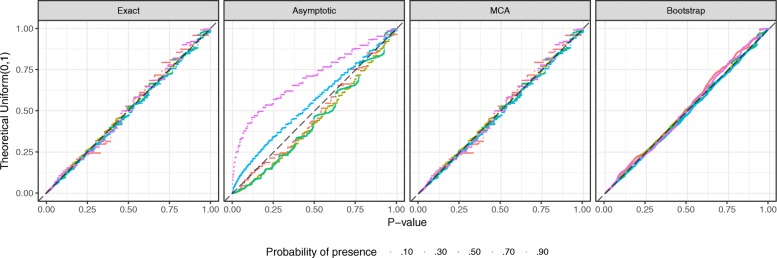
P-values of similarity among independent presence-absence vectors of *m*=100, with a wide range of probabilities *p*=.1,.3,.5,.7,.9. In each scenario, 2000 independent variables are simulated and tested using four proposed methods. The diagonal lines indicate a theoretically correct Uniform(0,1) distribution

First, we conducted 5 simulation scenarios using different underlying occurrence probabilities *p*=0.1,0.3,0.5,0.7,0.9 to generate independent presence-absence datasets. In essence, they are two species of length *m*=100 that exhibit unrelated co-occurrence patterns, where a proportion of presence (1’s) ranges from 10% to 90%. For each of simulation scenarios, a total of 2000 comparisons were made using a length *m*=100. Without any information about simulation parameters, our proposed methods are applied on an identically simulated dataset (Fig. 2). Theoretically correct *p*-values under the null hypothesis (null *p*-values) should form a Uniform distribution between 0 and 1, which are denoted by dashed diagonal lines in QQ plots. An upward deviation from diagonals shows an anti-conservative bias, as shown among some asymptotic *p*-values. In all scenarios, *p*-values from the exact solution, bootstrap (*B*=500), and measure concentration (accuracy =1×10^−5^) algorithms follow a theoretically correct Uniform(0,1) distribution (Fig. 2). Asymptotic approximation is inconsistent; its behavior is anti-conservative with *p*=0.3,0.5 and slightly conservative with *p*=0.7,0.9. Asymptotic approximation should only be used when computational time is a critical bottleneck.

**Fig. 3:**
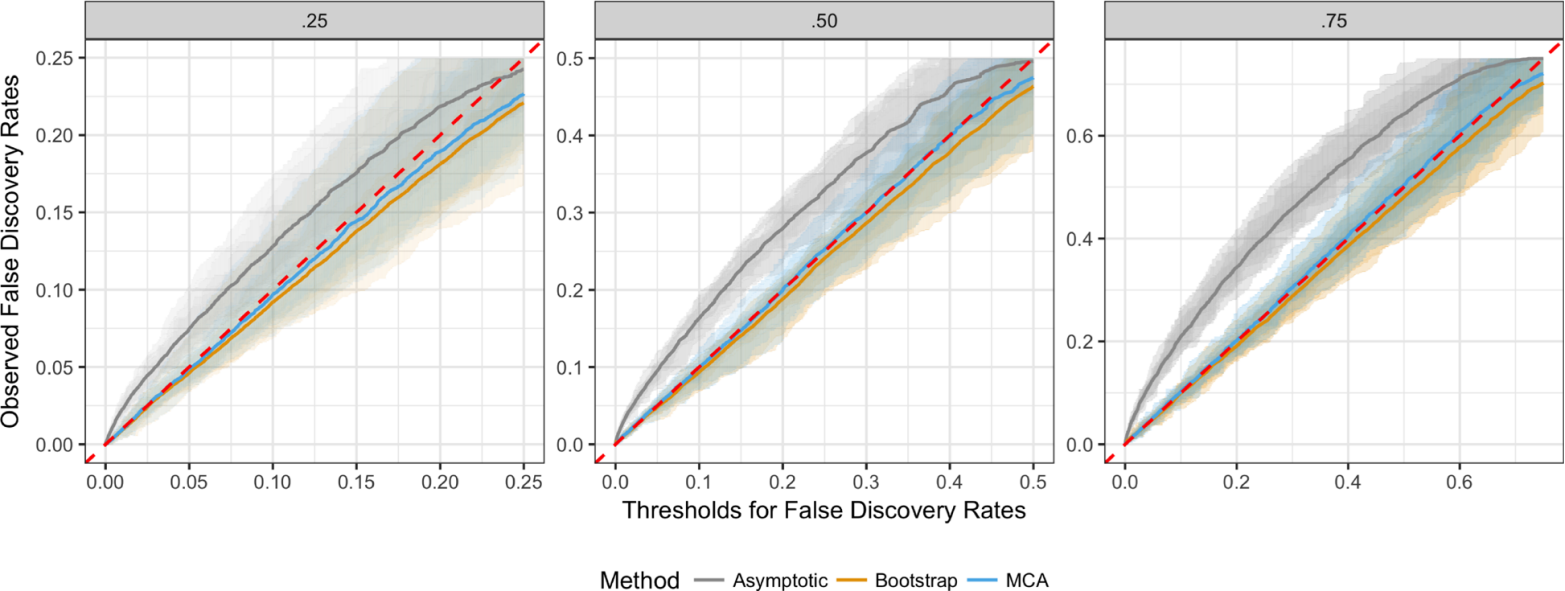
False discovery rate (FDR) estimates from a mixture of independent and dependent presence-absence vectors. In 3 separate scenarios with null proportions *π*_0_=.25,.50,.75, 2000 presence-absence vectors of *m*=200 are simulated with occurrence probabilities of *p*=.5. Each simulation scenario is repeated 20 times and the proposed methods are used to automatically compute *p*-values and q-values. FDR thresholds are plotted against observed false discovery proportions, where a downward deviation from a theoretically correct diagonal red line indicates a conservative behavior

Second, we generated a mixture of independent and dependent datasets out of *n*=2000 presence-absence vectors (of *n*=2000 species observed in *m*=200 biogeographic units) to evaluate false discovery rates. In three separate scenarios, we simulated 25%, 50%, and 75% of *n*=2000 species to be independent, resulting in null proportions of *π*_0_=.25,.50,.75 respectively. For example, a scenario with *π*_0_=.75 produces 500 out of *n*=2000 presence-absence variables that are truly associated with the query variable. Then, our proposed asymptotic approximation, bootstrap method, and MCA are used to automatically compute *p*-values. To account for variation in simulation, we repeated each scenario 20 times. FDRs and *π*_0_ are estimated by the q-value methodology [33]. Q-values are evaluated against FDR thresholds, so that we can evaluate accuracy of observed FDRs (Fig. 3). Twenty simulation replications are shown in semi-transparent shades, whereas their group averages for 3 methods are shown as solid lines. An upward deviation as shown by asymptotic approximation indicates an overall anti-conservative behavior, likely due to *m*↛*∞*. The bootstrap and MCA maintain the overall error rates, where the bootstrap exhibits slightly conservative characteristics (Fig. 3).

**Fig. 4:**
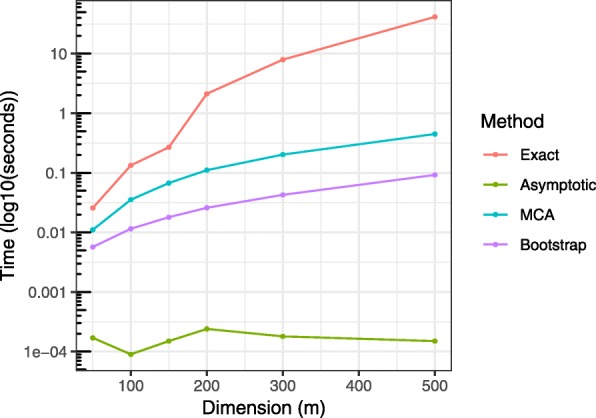
Computational runtimes of our 4 proposed methods. The means of 100 independent runs are plotted against an increasing size of dimension *m*=50,…,500. Compared to the exact solution, the bootstrap and measure concentration algorithm (MCA) provide vast improvements in speed whose relative efficiency increases with higher dimension

Third, we compared the computational efficiency of our proposed methods using our jaccard package on RStudio Cloud (Intel Xeon 2.90GHz and 1GB RAM), with R 3.5.0. We measured the runtime for a range of lengths *m*=50,…,500. For each *m*, we applied the proposed methods 10 times, with the bootstrap iteration *B*=5×*m* and MCA accuracy of 1×10^−5^. The average runtimes are shown Fig. 4. Our proposed computational methods show drastic improvement over the exact solution as *m* increases. The asymptotic approximation is mostly instantaneous. When the similarity between two presence-absence vectors of length *m*=500 were tested using the jaccard package, the exact solution was prohibitively slow, taking 41.5s on average. The bootstrap method was 449.8 times (0.09s) faster, whereas MCA was 92.5 times (0.45s) faster than the exact solution. Furthermore, we compared the runtimes of estimation methods for *m*=1000,…,10000 (Additional file 1: Figure S1). The gain in computational efficiency is more pronounced as the dimension (i.e., a length of presence-absence vectors) grows in size.

Last, a simulation study with *p*=0.5 and *m*=200 was used to evaluate two recent methods of species co-occurrences analysis. We generated independent presence-absence data where two species are truly unrelated. Then, methods of combinatorics [24] and hypergeometric distributions [25] are applied to obtain *p*-values. We followed the recommendations given in each paper, displaying four possible *p*-values from [24] (Additional file 2: Figure S2) and two one-sided *p*-values from [25] (Additional file 3: Figure S3). We observe these *p*-values under the null hypothesis to substantially deviate from theoretically correct Uniform(0,1) distributions.

### Applications in species co-occurrences

To show applications in statistically testing biological presence-absence data, the proposed methods are applied to species co-occurrence data. We investigated bird species on 28 islands in the Republic of Vanuatu, that are available in [6] and analyzed in several pioneering studies in non-random co-occurrences of species [12, 14–16]. The data is consisted of presence and absence of bird species in 28 islands of Vanuatu, which used to be known as the New Hebrides. Three generalist species that existed in all 28 islands were removed from our analysis. We are interested in identifying what pairs of species exhibit statistically significantly co-occurrences.

**Fig. 5:**
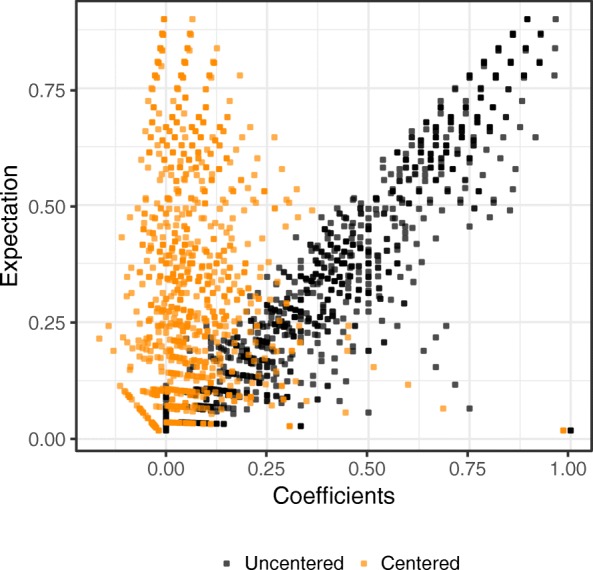
Comparison of uncentered and centered Jaccard/Tanimoto coefficients from the bird dataset. The conventional uncentered coefficients are shown to be strongly dependent on expectation under independence. By centering each coefficient by its expectation, the proposed centered coefficients alleviate this dependency

For *n*=53 bird species in *m*=28 islands, we obtained 1378 pair-wise Jaccard/Tanimoto similarity coefficients. The conventional Jaccard/Tanimoto coefficients depends strongly on their expected values under independence (Fig. 5). Similarly, the conventional Jaccard/Tanimoto coefficients are substantially correlated with the proportion of occurrences, with a Pearson correlation of 0.43 (*p*-value <2.2×10^−16^). Relying only on similarity coefficients would miss non-random co-occurrences among bird species that live in a few islands (Additional file 4: Figure S4). Our proposed methods account for co-occurrences that would be expected under independence. Histograms of the uncentered and centered Jaccard/Tanimoto coefficients are compared in Additional file 5: Figure S5.

**Fig. 6:**
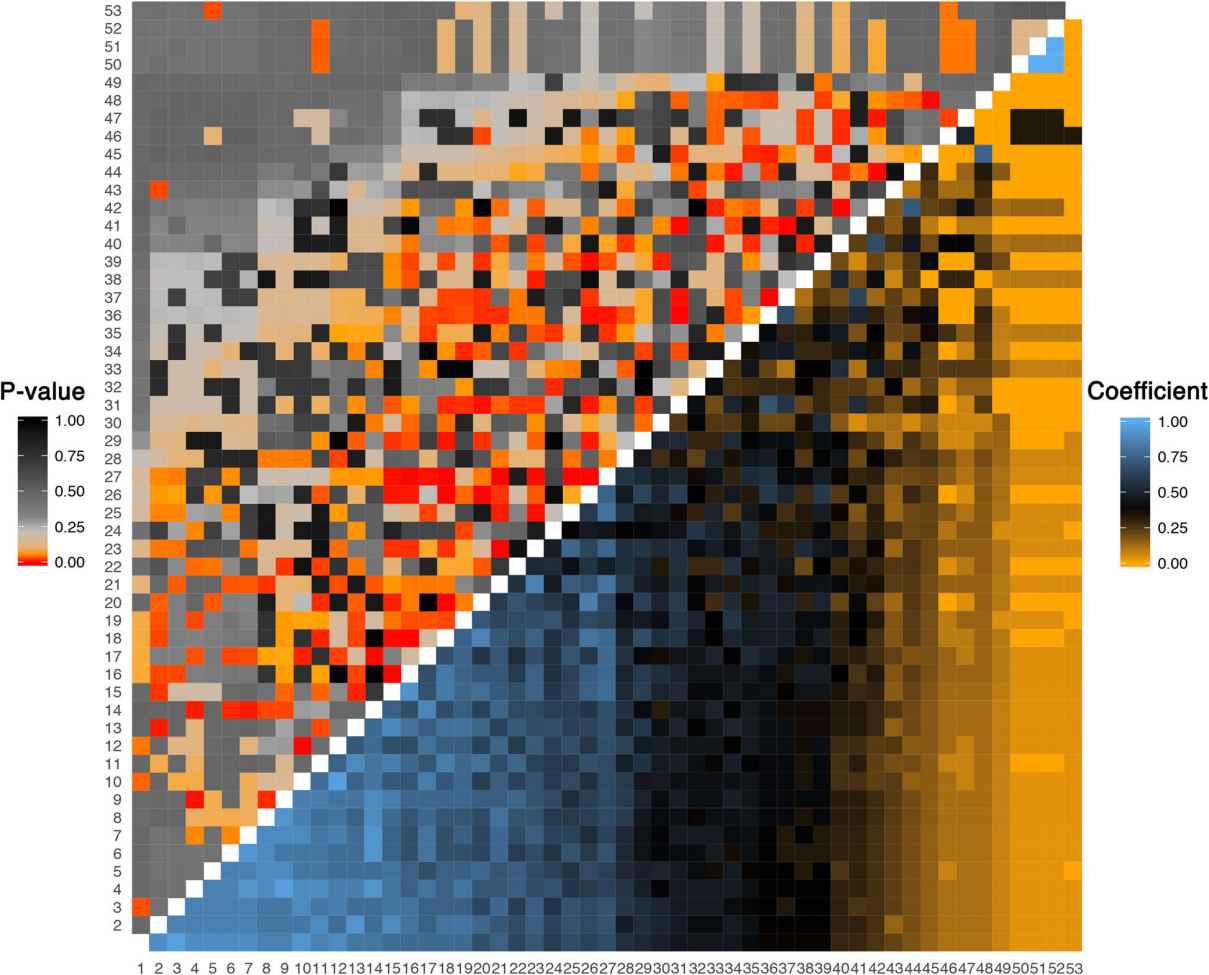
Heatmap of uncentered Jaccard/Tanimoto coefficients and their *p*-values. Similarity among 53 bird species in 28 islands of Vanuatu are tested using the proposed method. Species are ordered from high to low occurrences, that are highly correlated with Jaccard/Tanimoto coefficients (*p*-value <2.2×10^−16^). The upper triangle shows the *p*-values from our methods, whereas the lower triangle the observed Jaccard/Tanimoto coefficients

We computed statistical significance by applying the bootstrap method with *B*=5000 and MCA with accuracy of 1×10^−5^. Our two computational approaches estimated *p*-values that are almost identical with their mean squared deviation of 1.15×10^−4^ (Additional file 6: Figure S6). Significant results that are substantially deviating from random samples indicate non-random co-occurrences of species (Fig. 6). Out of 1378 pairs of species that were tested, the proportion of independent specie pairs was estimated to be 24% using q-value methodology [33]. Then, we calculated FDRs from 1378 pair-wise *p*-values. We discovered that 374 (27%) pairs are deemed significant at a q-value threshold of 0.10.

Additionally, we applied the Jaccard/Tanimoto similarity tests among fish species in French freshwater streams, surveyed over a long period of time [34]. Briefly, the presence and absence data of the *n*=32 most common fish species in *m*=3347 sites across French rivers are obtained during 1980 - 1991 [34]. Our analysis estimates that about 84.3*%* of 496 pairs are estimated to be non-randomly co-occurring. As surveyed for over a decade across Fresh rivers and surrounding habitats, it is reasonable that many fish species are interacting or influenced by related climate conditions. There are 21 pairs of species with q-values >0.1 (corresponding *p*-values ranging from 0.637 and 0.969). For example, the centered statistics between *Pungitius pungitius* and *Cyprinus carpio* is 3.31×10^−4^, whereas that between *Pungitius pungitius* and *Lota lota* is −4.40×10^−4^. *P. pungitius* is a small fish species typically riding in thick submerged vegetation with the breeding season falling in April - July. *C. carpio* and *L. lota* are much bigger species and generally prefers a large body of water.

## Conclusion

From biogeography to microbiology, evaluating similarity among species and biogeographic units is fundamental to assessing co-existence and biodiversity. Having observed occurrences of species in multiple biogeographic units, one of the primary goals in analyzing presence-absence data is to identify non-random co-occurrences. Even if two species would be present independently of each other, they may occur together by chance. For the last 30 years, the Jaccard/Tanimoto coefficient has been shown to be highly useful for quantitative analysis of co-occurrences that help inform systematic relationship among species [3–5]. We have developed a rigorous statistical framework and methods to efficiently calculate statistical significance of such similarity and to identify non-random co-occurrences.

For testing co-occurrences using the Jaccard/Tanimoto coefficient, we introduce exact and asymptotic solutions, as well as bootstrap and measure concentration algorithm. The proposed suite of statistical methods can provide a rigorous guideline to identify related species. Through comprehensive simulation studies, we characterized their operating characteristics using *p*-values and FDRs. The proposed bootstrap and measure concentration algorithms are highly accurate and efficient, providing orders of magnitude improvement in a computational speed. We have implemented the proposed methods in an open source R package and a Shiny web app. A user can upload a dataset to be analyzed, and create histograms and heat maps automatically. This will facilitate adaptation of *p*-values, FDRs, and related quantities in analyzing species co-occurrences.

Beyond species co-occurrences, the Jaccard/Tanimoto coefficient is used in diverse areas of biological science where binary data are observed and compared. When molecules and reactions are represented as hashed fingerprints, it is used for quantitative comparisons and classifications [35–37]. Similarity between biochemical reactions can be tested by applying our methods on their corresponding fingerprints. In genomics, the standard tools such as BEDTools [38] evaluate genomic intervals using the Jaccard/Tanimoto coefficients. Given genomic intervals from two samples or groups, one could test whether their overlap is statistically significant, providing evidences for shared genomic variations. Due to the popularity of Jaccard/Tanimoto coefficients, the proposed suite of methods would be useful in a broad range of scientific applications.

## Supplementary information

**Additional file 1** Computational runtimes when testing similarity between presence-absence data upto *m*=10000. We ran the proposed 4 methods to compute p-values for a wide range of dimension *m*. For each *m*, 100 independent simulations are conducted. Note that for *m*≥1000, the exact solution did not compute in a reasonable time. The bootstrap and measure concentration algorithm (MCA) are orders of magnitude faster than the exact solution. The asymptotic solution is instantaneous regardless of *m*.

**Additional file 2** Combinatoric p-values of similarity among independent presence-absence vectors of *m*=200 with *p*=.5. In each scenario, 2000 independent variables are simulated and tested using a combinatorics [24]. [24] recommends p_lt_+p_et_ and p_gt_+p_et_ as p-values. The dashed red lines indicate theoretically correct Uniform distributions.

**Additional file 3** Hypergeometric p-values of similarity among independent presence-absence vectors of *m*=200 with *p*=.5. We used a hypergeometric distribution [25] to obtain p-values of similarity between independent species. The original authors suggested that p_gt_ and p_lt_ can be “interpreted and reported as p-values”. The dashed red lines indicate theoretically correct Uniform distributions.

**Additional file 4** Scatterplot of marginal occurrences of 53 bird species and Jaccard/Tanimoto coefficients. As expected, we observe high correlation (Pearson correlation =0.43) between marginal occurrences and Jaccard/Tanimoto coefficients.

**Additional file 5** Histograms of conventional and centered Jaccard/Tanimoto similarity coefficients. The conventional (uncentered) Jaccard/Tanimoto coefficients are centered by their expected values under the independence assumption.

**Additional file 6** Comparison of p-values from the bootstrap and measure concentration algorithm (MCA). Both algorithms were applied on 1378 co-occurrences of bird species. The difference between estimated p-values from two methods is minimal with a mean squared deviation of 1.15×10^−4^. The diagonal red line indicates the identity.

## Data Availability

The jaccard package is available on the Comprehensive R Archive Network (CRAN) https://CRAN.R-project.org/package=jaccard, whereas the development version on Github https://github.com/ncchung/jaccard. The Shiny app is available at https://nnnn.shinyapps.io/jaccard/.

## Abbreviations

FDR: False discovery rate
i.i.d: Independent and identically distributed
MCA: Measure concentration algorithm
OTU: Operational taxonomic unit
