# Supplementary figures and images for: Jaccard/Tanimoto similarity test and estimation methods for biological presence-absence data
Source: BMC Bioinformatics. 2019 Dec 24;20(Suppl 15):644. doi: 10.1186/s12859-019-3118-5 (PMC6929325; doi:10.1186/s12859-019-3118-5)

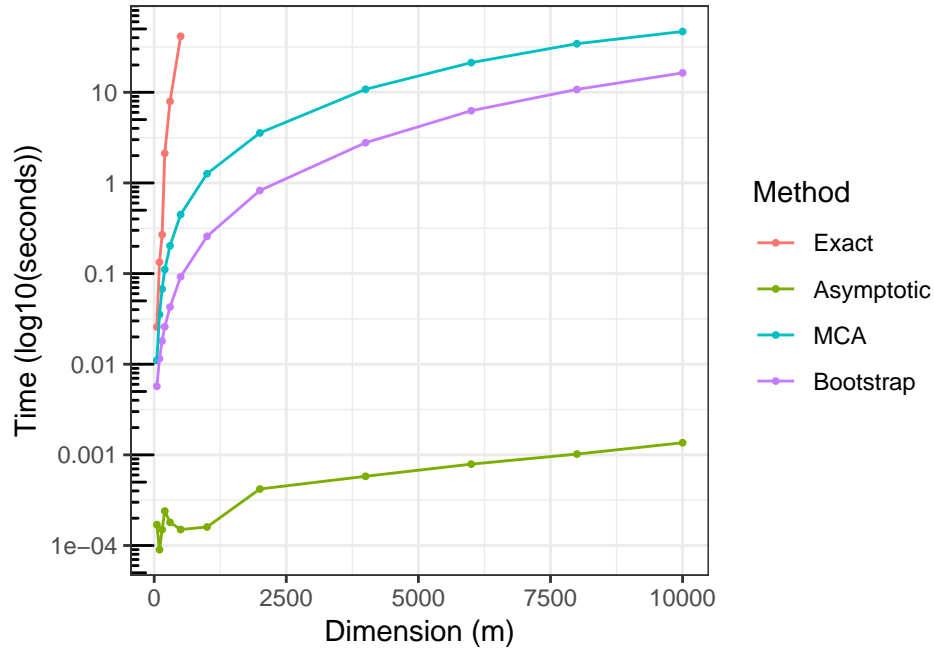

Supplement: Supplementary file 1 — Additional file 1 Computational runtimes when testing similarity between presence-absence data upto m=10000. We ran the proposed 4 methods to compute p-values for a wide range of dimension m. For each m, 100 independent simulations are conducted. Note that for m≥1000, the exact solution did not compute in a reasonable time. The bootstrap and measure concentration algorithm (MCA) are orders of magnitude faster than the exact solution. The asymptotic solution is instantaneous regardless of m. [file 12859_2019_3118_MOESM1_ESM.pdf]

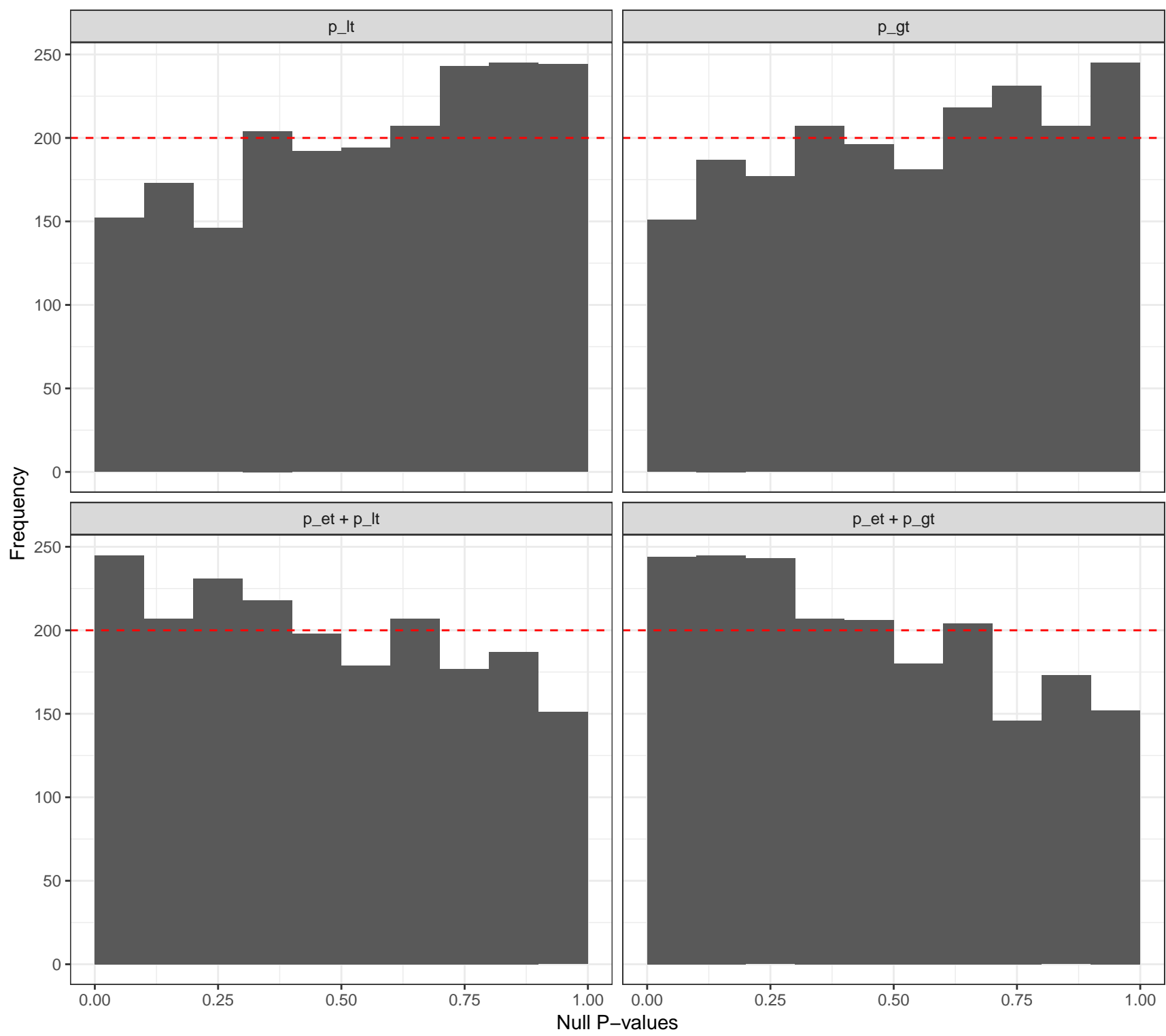

Supplement: Supplementary file 2 — Additional file 2 Combinatoric p-values of similarity among independent presence-absence vectors of m=200 with p=.5. In each scenario, 2000 independent variables are simulated and tested using a combinatorics [24]. [24] recommends plt+pet and pgt+pet as p-values. The dashed red lines indicate theoretically correct Uniform distributions. [file 12859_2019_3118_MOESM2_ESM.pdf]

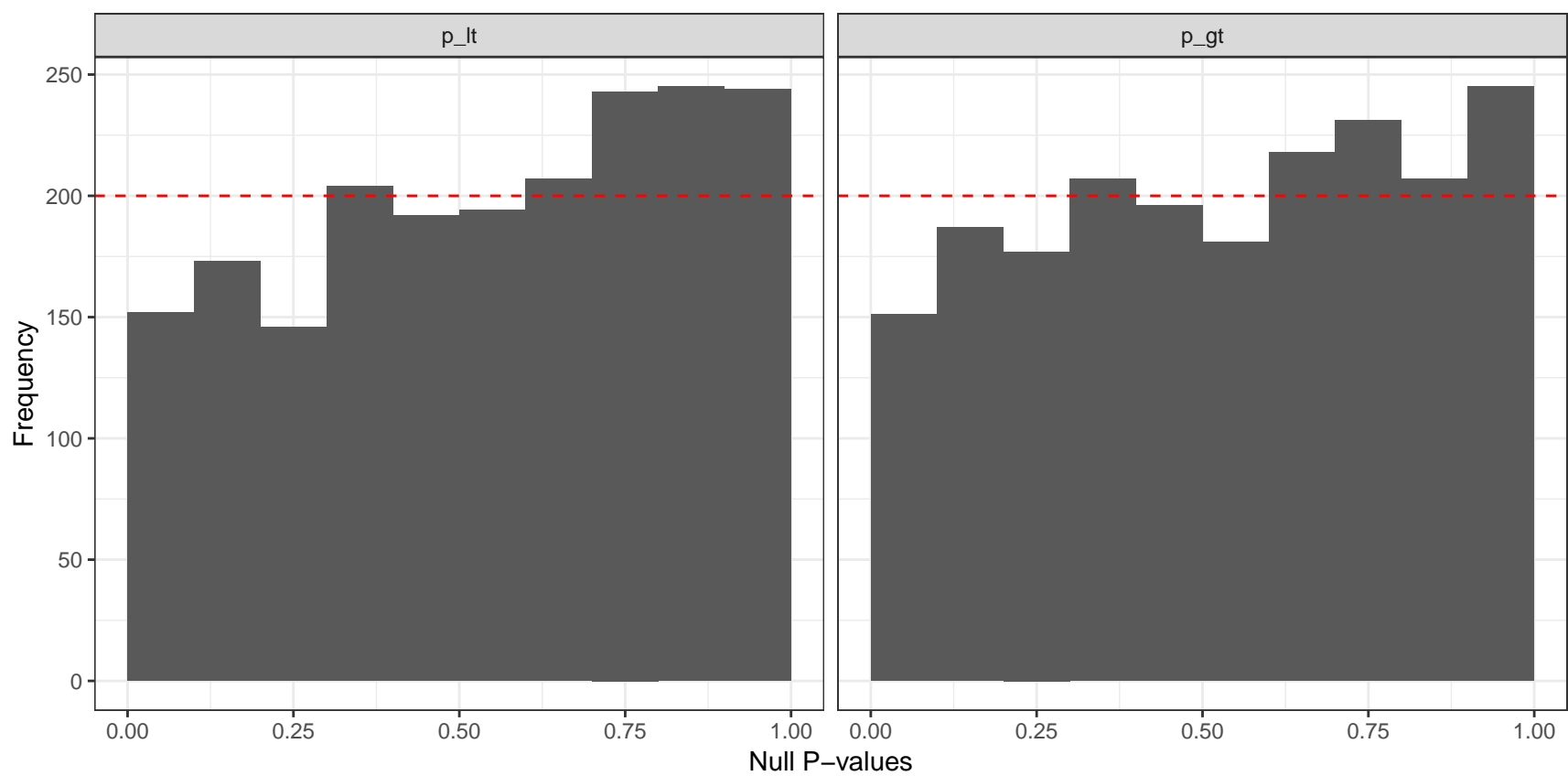

Supplement: Supplementary file 3 — Additional file 3 Hypergeometric p-values of similarity among independent presence-absence vectors of m=200 with p=.5. We used a hypergeometric distribution [25] to obtain p-values of similarity between independent species. The original authors suggested that pgt and plt can be “interpreted and reported as p-values”. The dashed red lines indicate theoretically correct Uniform distributions. [file 12859_2019_3118_MOESM3_ESM.pdf]

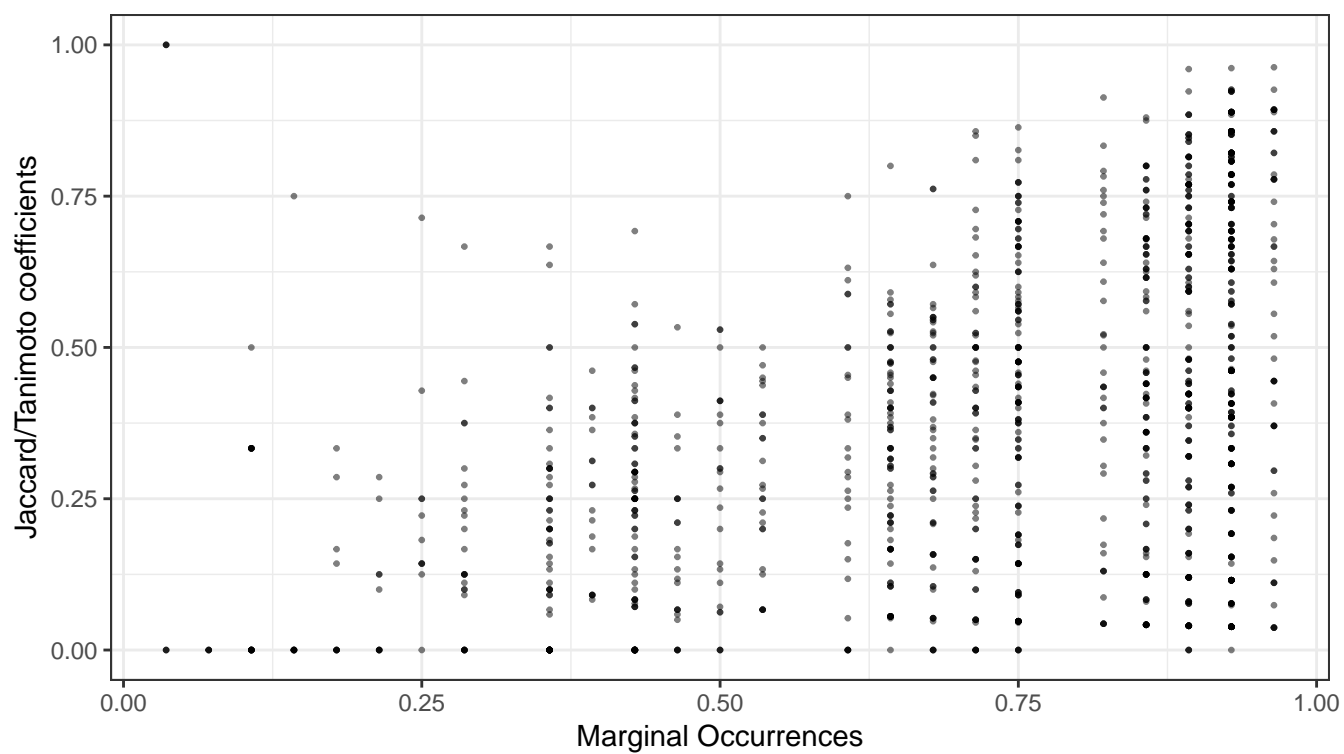

Supplement: Supplementary file 4 — Additional file 4 Scatterplot of marginal occurrences of 53 bird species and Jaccard/Tanimoto coefficients. As expected, we observe high correlation (Pearson correlation =0.43) between marginal occurrences and Jaccard/Tanimoto coefficients. [file 12859_2019_3118_MOESM4_ESM.pdf]

(a) Jaccard/Tanimoto coefficients

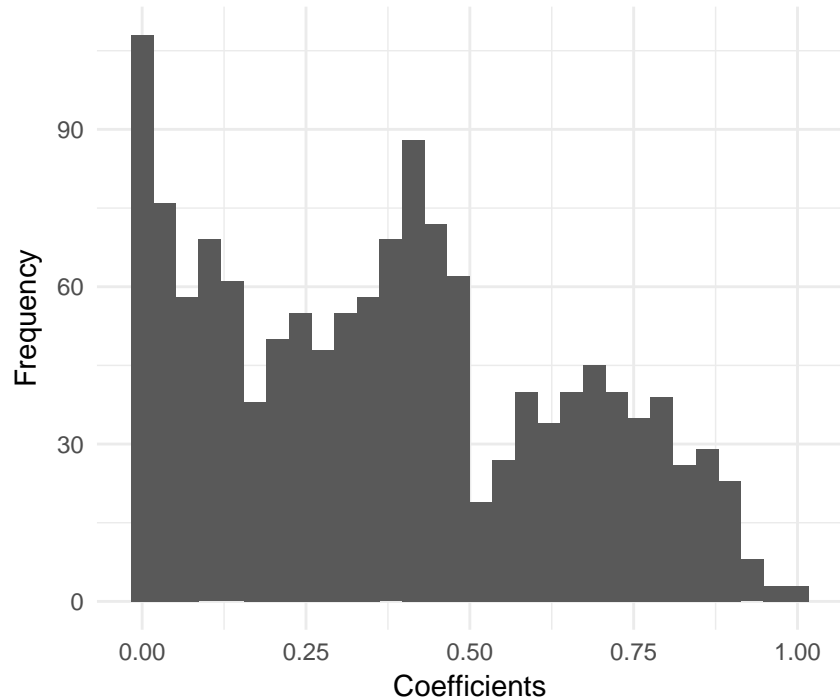

(b) Centered Jaccard/Tanimoto coefficients

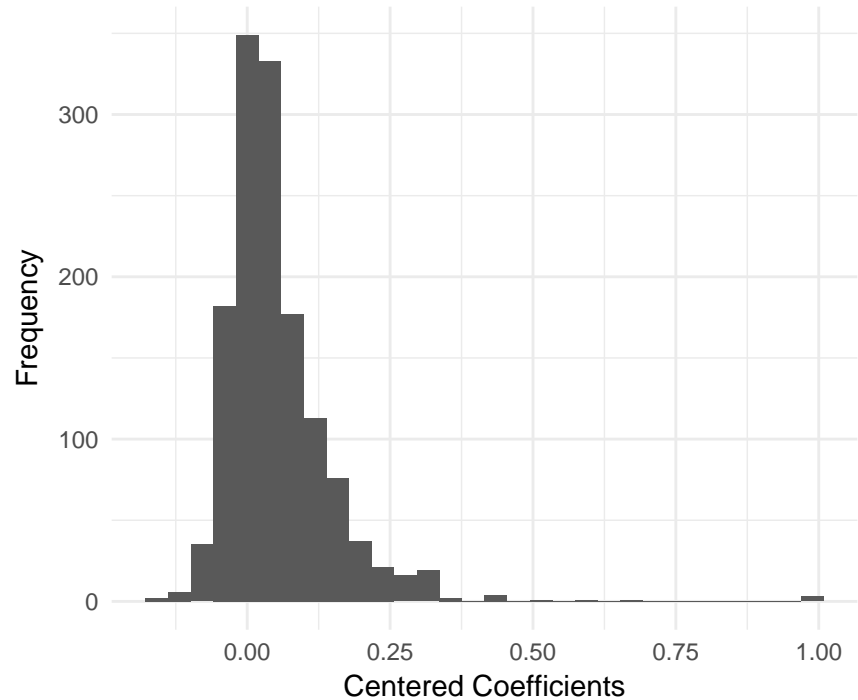

Supplement: Supplementary file 5 — Additional file 5 Histograms of conventional and centered Jaccard/Tanimoto similarity coefficients. The conventional (uncentered) Jaccard/Tanimoto coefficients are centered by their expected values under the independence assumption. [file 12859_2019_3118_MOESM5_ESM.pdf]

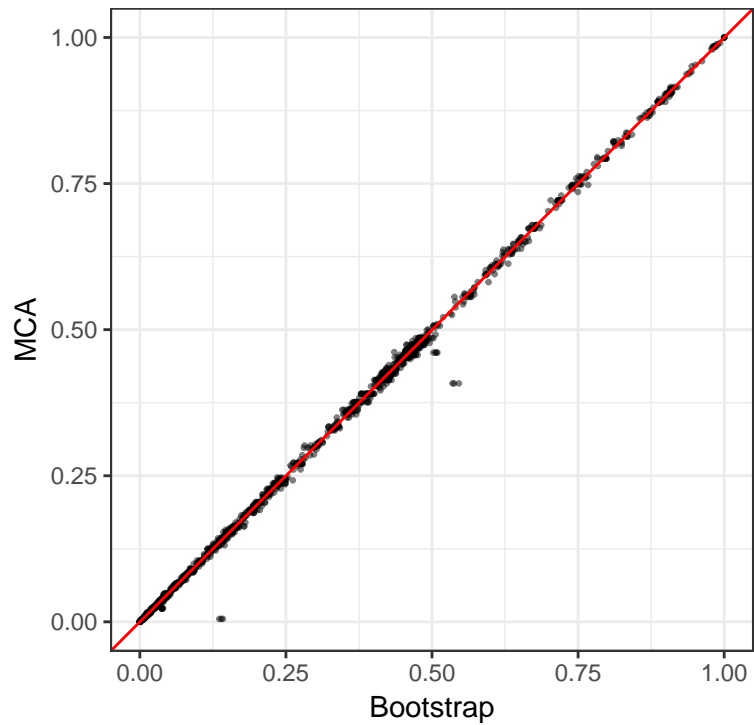

Supplement: Supplementary file 6 — Additional file 6 Comparison of p-values from the bootstrap and measure concentration algorithm (MCA). Both algorithms were applied on 1378 co-occurrences of bird species. The difference between estimated p-values from two methods is minimal with a mean squared deviation of 1.15×10^−4^. The diagonal red line indicates the identity. [file 12859_2019_3118_MOESM6_ESM.pdf]
